# Breakdown and reform: the Chilean road to the creation of ministries of hygiene and social welfare 1892–1931

**DOI:** 10.1017/mdh.2024.2

**Published:** 2024-07

**Authors:** Diego Barría Traverso, Diego Romero Pavez

**Affiliations:** 1 Universidad de Valparaíso, Gran Avenida José Miguel Carrera 4160, San Miguel, Santiago, Chile; 2 Pontificia Universidad Católica de Chile, Avda, Vicuña Mackenna 4860, Macul, Santiago, Chile

**Keywords:** Public health, Health ministries, Administrative reform, Administrative history, Latin America, Chile

## Abstract

Doctors have played an important role in the development of health institutions in Latin America. However, they are not the only profession that has had a voice in these matters. There are also other factors influencing the development of ministries of health. This issue has gone unnoticed in the literature. This article suggests that it is possible to identify two distinct trends in the creation of health ministries in Latin America. The first, of an early nature, was seen principally in Central America and the Caribbean in countries dependent on or under the influence of the United States which, from the 1880s, promoted health Pan-Americanism. The second trend, which became apparent from 1924, was characterised by the emergence of ministries in a context of institutional breakdown and the appearance of new actors (military or populist leaders). This second trend was first seen in Chile in 1924. This article analyses the creation of the Ministerio de Higiene, Asistencia y Previsión Social (Ministry of Hygiene, Assistance and Social Security) in Chile in 1924 and its subsequent development through to 1931. The analysis looks at the health measures adopted, the context in which this occurred and the debates triggered by the ministry’s process of institutional development, based on parliamentary discussions, presidential speeches, official statistics, legislation, documents prepared by key actors and the press of the time.

## Introduction

Doctors have played an important role in the development of health institutions in Latin America. Starting in the second half of the nineteenth century, health professionals in different countries began to form organisations and were an important factor in the progress of the region’s public health systems.^1^ However, they are not the only profession that has had a voice in these matters. In Chile and Ecuador in 1924 and 1925, respectively, young military officers also played a role in the development of the institutional framework. Chile’s September Revolution and Ecuador’s July Revolution opened the way to the creation of these countries’ first ministries responsible for health.

This issue has gone unnoticed in the literature, despite the existence in Latin America of an active community of historians who have sought to understand the development of both medicine and health systems in the region’s countries. As Espinosa showed,^2^ much of this work has focused on three topics: the development of health institutions in different countries; doctors’ participation in the expansion of medicine and their influence on public policy; and the role of overseas actors in the progress of public health in Latin America. Although Espinosa criticises the predominance of methodological nationalism,^3^ some work, such as that of Cueto^4^ and Birn,^5^ has provided a more detailed picture and identified clear stages in the development of health care, including a regional overview. One branch of the literature has focused on the rise of state intervention.^6^ Thanks to this work, we know that, from the 1880s, different countries began to establish government health departments that, in the 1930s and 1940s, became ministries. The creation of these departments has been explained as a response to socioeconomic changes and the conflicts arising from the modernisation of the latter part of the nineteenth century. Some studies have also drawn attention to the importance of political factors in the emergence and expansion of a state role.^7^

Although these works have described in detail the different stages of state intervention, some aspects require further study. For example, the literature has not explained why health departments, which reported to the ministries of interior,^8^ subsequently became ministries. Similarly, it is unclear why some countries embarked on this process before the 1930s and 1940s and in what specific contexts policymakers opted to reorganise their country’s public health institutions. What contextual factors were involved? Are there common patterns among countries, or, on the contrary, is it possible to identify diverse trajectories within the region?

This article addresses these questions based on an analysis of the creation of the *Ministerio de Higiene, Asistencia y Previsión Social* (Ministry of Hygiene, Assistance and Social Security) in Chile in 1924 and its subsequent development through to 1931. As shown in the next section, it is possible to identify two distinct trends in the creation of health ministries in Latin America. The first, of an early nature, was seen principally in Central America and the Caribbean in countries dependent on or under the influence of the United States which, from the 1880s, promoted health Pan-Americanism. The second trend, which became apparent from 1924, was characterised by the emergence of ministries in a context of institutional breakdown and the appearance of new actors (military or populist leaders). This points to something not noted in previous work: although the creation of health departments was not necessarily controversial, the establishment of ministries was problematic since it implied a change in the structure of the state and its role vis-à-vis society.

This second trend was first seen in Chile in 1924. This case is important because detailed analysis shows that the country’s first health ministry could be established because of the institutional breakdown Chile experienced that year. It marked the end of the political regime of the nineteenth century, opening the way for new actors who, in line with the ideas of the so-called September Revolution, gave state action a new face. However, this process was not without its difficulties and contradictions. For example, certain actors in Congress opposed the new ministry on the grounds of fiscal discipline, a longstanding concern in Chile. This led to difficulties in the ministry’s implementation. At the same time, the new ministry had to resolve fundamental questions about the reasons for its existence. Both the Ministry of Hygiene, Assistance and Social Security and the *Ministerio de Bienestar Social* (Ministry of Social Welfare), which replaced it in 1927, brought a series of departments related to this field under their command. This changed somewhat under President Carlos Ibáñez del Campo (1927–1931) who, through the Ministry of Social Welfare, tried to conceptualise a notion of welfare that, for a brief period, incorporated other spheres of intervention that the authorities considered crucial for the population’s quality of life. As regards the reasons for establishing a ministry, one option is to interpret it as reflecting the state’s unstoppable advance over society. However, this is not entirely convincing since the 1920s was a period of tension during which the actors held different views on how to organise the state.

This paper addresses these issues through process analysis that focuses on the creation of the Ministry of Hygiene, its development and transformation into the Ministry of Social Welfare between 1924 and 1931. The analysis looks at the health measures adopted, the context in which this occurred and the debates triggered by the ministry’s process of institutional development, based on parliamentary discussions, presidential speeches, official statistics, legislation, documents prepared by key actors and the press of the time.

The next section discusses the two different trajectories for the creation of health ministries observed in Latin America, emphasising the pioneering nature of the Chilean experience in inaugurating the era of ministries created as a result of major political changes. The following three sections examine the events that led to the establishment of the Ministry of Hygiene and the Ministry of Social Welfare in Chile, considering the situation prior to 1924, the effects of the September Revolution on the organisation of these ministries. Finally, the conclusions of the study are presented.

## Health ministries in Latin America: between the influence of the United States and the break with old regimes

For much of the nineteenth century, health in Latin America was a concern only partially addressed by the state. Health was a field in which philanthropic action and Christian charity played a central role.^9^ This began to change towards the end of the century in response to the pressure of modernisation processes. Precarious sanitary conditions were reflected in the appearance of a series of health problems, and there was growing discussion about living conditions. At the same time, there was pressure to ensure health conditions in shipping, and this quickly influenced the situation on land.^10^

According to García,^11^ Latin American states’ interest in medicine as a way to reduce tensions within society became apparent in the 1880s. As a result, ‘bureaucratic bodies are created which, with the name of directorates, departments or national services, are responsible for carrying out activities in the field of health’.^12^ From then on through to the 1930s, centralised units began to replace collegiate bodies. These initiatives sought to combat yellow fever in ports.^13^ State health or, in other words, direct government action began to replace charity,^14^ marking the start of a path, which would crystallise fifty years later, under which the liberal paradigm was replaced by that of social medicine where the state must play a preponderant role in establishing health systems.^15^

The literature on health systems in Latin America tends to highlight the appearance of a hygienist movement in various countries as one of the factors behind this phenomenon. This movement sought to influence public policy and improve sanitary conditions for the population through disease control and health education initiatives.^16^ Hygienism was facilitated by the existence of educational institutions in countries such as Cuba, Brazil and Argentina.^17^ In Chile, doctors played a fundamental role from the 1880s, proposing initiatives to combat cholera and smallpox.^18^ In Uruguay, public health professionals like Pedro Visca promoted medical knowledge and sought to influence health policies.^19^

Intervention in health was not only a concern within countries. Sea trade was a source of both progress and the spread of diseases. The traditional response to these situations – quarantine – had economic drawbacks. Starting in the 1880s, the United States began to promote sanitary Pan-Americanism.^20^ At the Second International Conference of American States, which took place in Mexico City between October 1901 and January 1902, the agreements reached included cooperation on health matters. This led to the creation of the Pan American Health Organization (PAHO) which, between 1903 and 1911, organised five meetings to coordinate measures to combat diseases such as yellow fever, malaria, bubonic plague and tuberculosis; reform ports’ quarantine system and ensure ready notification of outbreaks of epidemics that could be harmful to trade.^21^

The creation of the Rockefeller Foundation in 1913 also contributed to the dissemination of ideas in favour of state intervention in health.^22^ It focused on transferring resources for investment in health initiatives, training medical personnel at US universities and fostering awareness of scientific-technological advances as well as directly advising a number of countries such as Puerto Rico, Costa Rica, Guatemala, El Salvador, Brazil and Colombia.^23^

García suggests the era of state medicine was inaugurated in Argentina with the creation of the *Departamento Nacional de Higiene* (National Department of Hygiene) in 1880.^24^ Similar measures followed in Chile (1889),^25^ Uruguay (1895), Brazil (1897), Paraguay (1899), Peru (1903), Bolivia (1906), Guatemala (1906), Ecuador (1908) Venezuela (1911) and Colombia (1918).^26^ But it was not until the 1920s, that countries began to create ministerial-level bodies for public health. The exception was Cuba which, in 1909, created the *Secretaría de Sanidad y Beneficencia* (Health and Charity Secretariat).^27^ In 1917, in Mexico an era of social constitutionalism was inaugurated, but it did not immediately move to establish a ministry in charge of health. Indeed, the literature tends to highlight a continuity with the approach adopted during the Porfiriato.^28^ A ministerial-level body was not established until 1938 when the *Secretaría de Asistencia Social* (Social Assistance Secretariat) was formed.^29^

As Table 1 shows, until the 1920s, only a few countries had established a ministerial-level organisation for the health sector. Cuba was followed by the Dominican Republic, which established a *Secretaría de Sanidad y Beneficencia* (Health and Charity Secretariat) in 1920 while, in 1923, El Salvador established a Ministry of Health and Charity.^30^ This limited action was despite the signing of a Pan-American Health Code at meetings organised by PAHO in Havana (1924) and Lima (1927), which recognised health as a right of all citizens and established commitments for states’ implementation of health policies through their respective ministries.^31^

**Table 1.** Creation of health ministries in Latin America, selected countries

Country	Year	First ministry with ‘health’ in its name	Government
Cuba	1909	Health and Charity Secretariat	José Miguel Gómez (elected after US occupation)
Dominican Republic	1920	Health and Charity Secretariat	Juan Bautista Vicini Burgos (during US occupation)
Colombia	1923	Education and Public Health Ministry	Pedro Nel Ospina (Conservative Party)
El Salvador	1923	Health and Charity Ministry	Alfonso Quiñónez Molina (National Democratic Party)
Chile	1924	Hygiene, Assistance and Social Security Ministry	Luis Altamirano Talavera (Government Junta)
Ecuador	1925	Ministry of Social Security, Labour, Agriculture, Welfare, Health, Hygiene, Statistics and Immigration and Colonisation	Government Junta
Costa Rica	1927	Public Health and Social Protection Secretariat	Ricardo Jiménez (Republican Party)
Brazil	1930	Education and Public Health Ministry	Getúlio Vargas (provisional government appointed by a military junta)
Uruguay	1934	Public Health Ministry	Gabriel Terra (coup)
Peru	1935	Public Health, Labour and Social Security Ministry	Oscar Benavides (head of army; appointed as provisional president)
Paraguay	1936	Public Health Ministry	Rafael Franco (coup)
Venezuela	1936	Health and Social Assistance Ministry	Eleazar López (soldier; appointed and then elected)
Bolivia	1938	Hygiene and Public Health Ministry	Germán Busch (*de facto* government)
Mexico	1938	Social Assistance Secretariat	Lázaro Cárdenas (National Revolutionary Party)
Argentina	1949	Public Health Ministry	Juan Domingo Perón (Justicialist Party)

*Source:* Compiled by authors based on Portillo, *op. cit.* (note 9); Delgado, *op. cit.* (note 16); Quevedo, *op. cit.* (note 21); Gregorio Mendizábal, *Historia de la Salud Pública en Bolivia. De las Juntas de Sanidad a los Directorios Locales de Salud* (La Paz, Organización Panamericana de la Salud, 2002); Bustamante (ed.), *100 años de salud*; Jairnilson Paim, Claudia Travassos, Celia Almeida, Ligia Bahia and James Macinko, ‘The Brazilian Health System: History, Advances and Challenges’, *The Lancet*, 377, 9779 (2011), 1778–97; Cueto *et al*., *op. cit.* (note 1); Biernat, *op. cit.* (note 17); García, *op. cit.* (note 6).

The literature has identified certain patterns that help explain the irruption of public health in Latin America. They include the transition and conflict that occurred from the 1880s,^32^ the role of doctors^33^ and the influence of the United States or international organisations.^34^ However, the reasons for creating ministerial-level organisations are less clear and Table 1 reveals a pattern that has been little explored: the existence of two different trajectories for the creation of health ministries.

The first is directly related to the influence of the United States, as indicated by García,^35^ and its presence in the Caribbean and Central America.^36^ Indeed, the first ministries were created in Cuba, the Dominican Republic, El Salvador and Colombia, all of which had a relationship of economic, political and military dependence and weakness with the United States. In the case of Cuba, the decision to create a ministry was made by President José Miguel Gómez, elected after the country’s occupation by the United States. Similarly, the Dominican Republic set up its ministry during the US occupation and at a time when the United States was the market for more than half of its exports and its only source of foreign investment.^37^ El Salvador, like the rest of Central America,^38^ was economically dependent on its main trading partner.^39^ Moreover, the US government sought to influence domestic political struggles, generating anti-American sentiment.^40^ From 1914, US relations with El Salvador were on a good footing,^41^ enabling this country to be one of those that received collaboration from the Rockefeller Foundation.^42^ At this time, Colombia was unable to defend its territorial integrity, losing Panama in 1903 as a result of direct US intervention.^43^ However, the Urrutia–Thompson Agreement, which put an end to this conflict and established due compensation,^44^ marked the start of an era of expansion of US economic presence in Colombia,^45^ accompanied by a fluid relationship on administrative matters. In 1917, Colombia received its first contributions from the Rockefeller Foundation which, at the request of the Colombian government, financed a US commission to study yellow fever in the country. Through to 1935, there were nine other examples of this collaboration.^46^ President Pedro Nel Ospina established the *Ministerio de Instrucción y Salubridad Pública* (Ministry of Education and Public Health) in the same year that he hired the services of US consultant Edwig Kemmerer.^47^ This first pattern in the creation of health ministries is also seen in Costa Rica. It had a fluid relationship with the United States in the health field to the point that the Rockefeller Foundation helped with the institutionalisation of the School Health Department in 1914 and the Hookworm Infection Department in 1915. These units paved the way for the creation in 1922 of the *Subsecretaría de Higiene y Salud Pública* (Hygiene and Public Health Undersecretariat) and the *Secretaría de Salubridad Pública y Protección Social* (Public Health and Social Protection Secretariat).^48^

The second road to the creation of a health ministry was inaugurated by Chile and Ecuador. It is characterised by an institutional breakdown and/or the emergence of a populist project in response to political, social and economic crises not resolved by the *ancien régimes* of the nineteenth century. This path became more common from the 1930s but, in Chile, the process began in 1924. As discussed in the next section, the so-called September Revolution and the July Revolution in Chile and Ecuador, respectively, have an element in common:^49^ a movement spearheaded by a group of young soldiers led to the formation of governments that sought to give the state a new role, marked by administrative reform that included the creation of ministries responsible for health.^50^

The importance of these political dynamics has not been adequately studied. Even the pioneering work of García does not consider them as an explanatory variable.^51^ This omission limits understanding of the history of public health in Latin America since, as experiences such as those of Chile and Ecuador seem to indicate, the health care provided by the state reflects a change in its role and, as Clark suggested,^52^ its relations with the different social classes.

In summary, the relationship between political breakdown and the creation of a health ministry shows that the establishment of lower-ranking departments can be a functional response to problems of a social nature. However, creating a ministry gives rise to a series of conflicts within society. In this sense, an in-depth understanding of the process of creation of Chile’s health ministry serves to identify some points that can subsequently be used to search for common patterns in other cases. In addition, as shown below, the growth of public health was far from following a pattern of continuous progress and, on the contrary, faced various difficulties related to political debates and national administrative traditions.

## The irruption of social debate and social institutions in parliamentary Chile

For much of the nineteenth century, Chile was a traditional society. Although the state made some efforts to develop hospital capacity,^53^ health problems were addressed mostly through charity and philanthropy.^54^ Starting in the 1880s, Chile was hit by a cholera epidemic, leading to the adoption of health measures.^55^ In parallel, urbanisation was growing, increasing from 29 percent in 1865 to 43 percent in 1895. In absence of state social protection, the urban proletariat began to establish mutual societies and voice labour demands.^56^ Until 1902, mobilisations were ‘exceptional’ but workers then began to mobilise more actively.^57^ The country’s elite feared a social revolt and the state used the army for repression.^58^

However, the use of force was not the only response. In various fields, such as law, there began to be discussion about how to address the growing social problems.^59^ The political parties began to incorporate social problems into their debates. In 1905, the conservatives, influenced by the *Rerum Novarum* encyclical of Pope Leo XIII, proposed a policy of ‘economic, social and moral improvement’ while, in 1907, the liberals advocated harmonising the interests of employers and workers to avoid class struggle. In 1906, the radicals focused their political action on improving the conditions of the poor while the democrats urged an expansion of education and civic rights.^60^

As a result of these changes, a framework of social institutions began to be formed, giving priority to health care and labour and housing issues. In the case of health care, state intervention dated back to the 1880s when it had begun to replace private initiative. New social problems and, particularly, the crisis caused by the arrival of cholera in December 1886 led the Balmaceda government to take hitherto unthinkable measures. This, in turn, resulted in the creation in 1889 of the *Consejo de Higiene Pública* (Public Hygiene Council) which, in 1892, became the *Consejo Superior de Higiene Pública* (Higher Public Hygiene Council).^61^ The *Instituto de Higiene* (Hygiene Institute) also opened in 1892 and trained the main doctors who were to play a fundamental role in the creation and operation of the Ministry of Hygiene: Alejandro del Río, Pedro Lautaro Ferrer and Lucio Córdova.^62^ At the same time, the state began to provide drinking water. These health institutions reported to the Ministry of Interior.

During the 1890s, the medical community expressed its support to the performance of both the *Consejo* and *Instituto.* ^63^ At the same time, they alerted authorities the necessity of introducing some reforms to the legal framework, improve infrastructure and hiring more personnel.^64^ Some of these proposals were heard by authorities. For example, in 1898, the post of health inspector was introduced into legislation,^65^ as the *Instituto* suggested three years before.

During the 1900s a consensus on the weakness of the Chilean state in dealing with epidemics. Consequently, the then minister of interior, Elías Fernández, announced a project aiming at creating health stations throughout Chile.^66^ In 1906, del Río raised the idea of transforming the Higher Public Hygiene Council transformation into a *Subsecretaría de Higiene y Asistencia Pública* (Hygiene and Public Assistance Undersecretariat).^67^ This did not happen but a Hygiene and Beneficence Section was created in the Ministry of Interior, with the objective of ‘standardizing the actions of the various authorities and corporations to which the laws confer the care of public health’.^68^

At the same time, a set of health providers was emerging. By 1909, Chile had ninety-eight hospitals of which thirty-six were state hospitals, forty-one reported to the Board of Charity three were municipal, five were privately owned and five were ecclesiastical while the ownership of the remaining five was not specified.^69^

In 1907, a Labour Office was created. Initially designed to gather information, it soon began to undertake other initiatives such as a project, presented in 1913, that sought to combine research with the exercise of supervisory powers. It also became a center for the preparation of social policy proposals on, for example, pensions.^70^

Urbanisation brought with it problems of housing availability and hygiene in popular sectors. The popular rooming houses, known as *conventillos*, were quickly identified as a problem, and, in 1903, Congress set up a commission to study the situation. Its work culminated with the 1906 Workers’ Housing Law and the establishment of councils with powers to demolish premises declared insanitary. This law also introduced benefits for housing that met hygiene standards and charged low rents. In addition, through the *Caja de Ahorro Obrero* (Workers’ Compensation Fund), workers were encouraged to save to buy these properties.^71^

Laws were gradually introduced on matters such as Sunday rest (1907), homeless children (1912), the right to sit down at work (1914), workplace accidents (1916) and nurseries (1917). These measures were not enough to resolve the social problems and, in the years that followed, pressure mounted for further intervention.^72^ In this context, the then minister of interior, Arturo Alessandri, promoted an agenda of social reforms in 1918, including primary education, work, alcoholism and women’s role in society.^73^

Different voices, both in and outside Chile, urged the need for greater state intervention in health and labour matters. In 1906, the Higher Public Hygiene Council initiated the preparation of the first health code, which was sent to Congress in 1910.^74^ In 1917, the Ministry of Interior regretted the nonapproval of the bill. According to the José Luis Sanfuentes government (1915–1920), they were not able to tackle efficiently health issues due to the lack of a health code.^75^ The medical community, which during the presidency of Sanfuentes was able to influence health policies on issues such as the combat of the typhus epidemic,^76^ expressed similar views.^77^

The need to introduce new laws and institutions in the field of public health was also constantly discussed at the different PAHO conferences and,^78^ in the face of the financial crisis, the First Public Charity Congress, held in 1917, raised the need for the Chilean state to adopt a preponderant role.^79^ Chilean Law N° 3.385, drafted by Lucio Córdova with the help of del Río and introduced in 1918, established the country’s first health code. It created the *Dirección General de Sanidad* (General Directorate of Health), whose first director was Ramón Corbalán Melgarejo, and the Higher Council of Hygiene. The Institute of Hygiene reported to the General Directorate of Health, which also had a number of supervisory powers (see Law N° 3.385). At the same time, an institutional framework for health was set up in the provinces. In other words, the Directorate was the first body to centralise the management of health and, according to Cruz-Coke,^80^ became a *de facto* ministry.

The medical community criticised the code. According to them, it was a legal framework, written by politicians in place of physicians. Consequently, the law approved by the Congress had many mistakes that could even cause deaths among the Chilean population.^81^

The health sector grew in terms of both providers and services (Table 2), based on a combination of the state’s role in charity and the services provided by mutual aid societies. However, the state was the main actor. For example, in 1917, state contributions accounted for two-thirds of public charity. It was, therefore, not surprising that there were ongoing calls for the sector’s reform, now with a systemic approach.^82^

**Table 2.** Hospitals, personnel, patients and beds, 1908–1924

	1908	1909	1910	1911	1912	1913	1914	1915	1916
Hospitals	87	98	97	98	n/a.	102	103	108	109
N° of patients attended	102 277	104 790	103 562	106 217	n/a.	109 896	109 858	110 976	117 560
N° of doctors	n/a	200	n/a	362	n/a.	n/a	n/a	n/a	n/a
Total personnel	n/a	n/a	n/a	3661	n/a.	n/a	n/a	n/a	n/a
N° of beds	n/a	n/a	n/a	10506	n/a.	n/a	n/a	n/a	n/a
	1917	1918	1919	1920	1921	1922	1923	1924	
Hospitals	109	110	114	116	109	117	118	117	
N° of patients attended	122 206	130 369	140 039	142 371	145 889	141 474	149 642	149 411	
N° of doctors	450	463	470	528	491	500	575	609	
Total personnel	3 929	4 256	4 155	4 014	4 613	4 769	4 860	5 042	
N° of beds	10 658	10 860	10 715	11 806	11 591	12 006	12 151	12 623	

* Doctors and other personnel include both external and internal personnel.

** Personnel initially corresponds to doctors, trainees and other personnel to whom dentists, nurses and chemists are subsequently added.

*** Beds includes both free and paid beds.

*Source:* Oficina Central de Estadística, *Anuario Estadístico de la República de Chile* (Santiago, Oficina Central de Estadística, 1908–1925)

In the case of labour issues, new legislation had been discussed since 1918 with the idea of establishing mechanisms for mediation between capital and labour. In this context, the creation of a department exclusively for labour matters was proposed and, in 1919, the government of Sanfuentes promoted the establishment of a special commission in Congress to discuss this proposal.^83^ Proposals were also put forward for greater state intervention in the field of housing. Workers’ housing councils had power only over the houses for rent and could not control conditions in other housing. Further problems arose as a result of an increase in rents due to the renovation of demolished buildings and migration to the city.^84^

During the 1920 presidential campaign, Arturo Alessandri, a liberal, proposed a government program similar to that outlined in 1918. He presented himself to public opinion as a threat to those who opposed social change, promising to take measures on labour, social security and housing. The latter was an issue with which he was particularly familiar since it was the subject of his thesis for qualification as a lawyer in 1893.^85^ Alessandri also aimed to reform Chile’s constitution. On taking office as president, he presented a labour code bill to the Chamber of Deputies but was unsuccessful in obtaining approval for his agenda. This was hardly surprising since these were the last years of what has been termed Chilean pseudo-parliamentarianism.^86^ Since 1861, certain practices had been established through which Congress was able to dominate the political game at the expense of the executive.^87^ Alessandri was not immune to these problems and had to reshuffle his cabinet eighteen times in 44 months.^88^

The Alessandri government continued collaborating with the medical community as did Sanfuentes. In 1920, the Higher Council of Public Welfare was created. Additionally, authorities discussed the possibility of reforming the institutional framework to centralise the management of the health sector. This new organisation would be a ministry or an undersecretary. According to the government, ‘the aphorism “to spend on hygiene is to economise” is the synthesis of the modern concept that statesmen must always bear in mind not only as a measure of public happiness but also as a cause of economy for the Treasury’.^89^

The medical community agreed with Alessandri’s government plan.^90^ The idea of creating a Ministry of Health and Social Assistance was discussed in 1921 by the members of the Radical Party and by the members of the *Sociedad Médica.* ^91^ In 1923, a bill was presented for the creation of a Ministry of Hygiene.^92^

In the congressional elections of 1924, the Liberal Alliance triumphed, suggesting that it would be able to move forward with its reform program. For this task, the Alessandri appointed Pedro Aguirre Cerda, who had headed his first cabinet in 1920, as minister of interior.^93^ By September, the social agenda showed little progress and, in the context of a delay in paying the salaries of public officials and members of the armed forces, Congress discussed the introduction of remunerations for members of Congress. In response, a group led by young army officers, including Carlos Ibáñez del Campo, Bartolomé Blanche and Marmaduque Grove, demonstrated in Congress on September 2 and 3, 1924, demanding approval of a series of social reforms and improvements in the conditions of public employees. This group of young soldiers viewed direct action by public institutions as a way to improve social conditions in the country.^94^

On September 5, Alessandri met with the young army officers, who had formed a junta. He had to modify his cabinet, incorporating representatives of the armed forces, with General Altamirano as his new minister of interior. On September 8, a package of social reforms was approved, including norms on contracts, compulsory insurance, workplace accidents, labour disputes, unions, cooperatives and private-sector employees. Alessandri expected that this would mean the end of military intervention in politics and, when this proved not to be the case, he resigned, opening the way for a junta headed by General Altamirano, supported by other high-ranking members of the armed forces.^95^

## The September Revolution and the creation of the Ministry of Hygiene

The events of September 1924 marked the end of Chilean parliamentarianism. Once he had formed his government, Altamirano closed Congress. The plan was to implement a series of reforms and call elections. On September 11, the young army officers published a manifesto setting out the movement’s ideas. They included:

CORRUPTION IN THE REPUBLIC’S POLITICAL LIFE HAS LED OUR INSTITUTIONS TO AN ABYSS IN WHICH THE CONSTITUTION ITSELF BEGINS TO SUCCUMB TO MERELY PERSONAL INTERESTS …

The misery of the people, speculation, bad faith on the part of the powerful, economic instability and a lack of hope of regeneration within the existing regime had produced a ferment that irritated the bowels of the classes whose struggle for life is more difficult….^96^

The manifesto also indicated that a constituent assembly would be called to address the situation.

This chain of events not only implied the end of a political regime; it also opened the way to direct state action in the social sphere. This is how the doctors appeared to interpret the situation since, only a few weeks after the installation of the government junta, the Santiago Assembly of Doctors proposed the creation of a Ministry of Hygiene, Public Assistance and Social Security. The idea was supported by leading doctors such as Roberto Aguirre Luco, the dean of the Faculty of Medicine of the University of Chile, as well as Alejandro del Río, Eugenio Cienfuegos and Hugo Lea Plaza.^97^ Indeed, a year earlier, del Río had given a series of talks to promote the idea of creating this new state body.^98^ After a meeting with Altamirano, the junta undertook to study the initiative and, on October 14, decreed the creation of a Ministry of Hygiene, Assistance, Labour and Social Security on the grounds of the country’s ‘need to improve our sanitary conditions (…) and reduce to the minimum possible the economic-social disturbances arising from our excessive mortality’.^99^

The junta appointed del Río as Chile’s first minister of hygiene and began to implement the new institutional framework. In the case of public health, a number of prominent members of the Santiago Assembly of Doctors played a fundamental role. The new ministry also incorporated the *Dirección General del Trabajo* (General Directorate of Labour), reorganised under Law N° 4.053, and the *Consejo de Habitación Obrera* (Workers’ Housing Council). According to the minister, their incorporation under a single body would make it possible ‘to put the numerous labour problems on their right path’.^100^

One of the first measures taken by Minister del Río was to secure international advice, asking the United States to send an expert in the field.^101^ The expert chosen was John D. Long, a US doctor who, at the beginning of the twentieth century, had advised the government of the Philippines and, in 1924, had drafted the Pan American Sanitary Code.^102^ Del Rio and Long were acquainted through the Pan-American conferences. On arriving in July 1925,^103^ Long set out to help with the organisation of health institutions. The *Sociedad Médica de Chile* was enthusiastic about this appointment.^104^

In an opinion column published in the press, Dr. Cienfuegos defended the Ministry’s creation, arguing that the conditions required for a task of this nature existed: well-prepared plans, capable technical personnel and the support of the authorities. In his opinion, the centralisation of previously separate functions promised that state investment ‘would yield far more significant results than those obtained so far’.^105^ The new ministry comprised three sections: Hygiene, Social Assistance and Social Security, and Labour. In December of the same year, the *Consejo Superior de Beneficencia* (Higher Council of Charity) was replaced by the *Consejo Superior de Asistencia Social* (Higher Council of Social Assistance).^106^

In the months following the installation of the junta, the political situation remained unstable. The Altamirano government had to coexist with the young army officers, who continued to be organised as a kind of guarantor of the ideology of the September 11 manifesto. In December, Altamirano managed to persuade the young men to dissolve their organisation in exchange for a modification of his cabinet. However, the young officers were not satisfied with the new cabinet and, in January 1925, overthrew the junta which, at this point, was already openly close to conservative sectors.^107^ A new government junta was established, led by Emilio Bello Codesio, and prepared to bring back Arturo Alessandri.

Del Río left the government together with Altamirano and was replaced by Dr. José Santos Salas, who began to reorganise the ministry. In January, housing tribunals were established and rents were regulated and a *Consejo Superior de Protección de la Infancia* (Higher Council for the Protection of Childhood) was set up to coordinate public and private services in this field. In February, the Ministry was divided into two undersecretariats: Social Hygiene, Public Health and Public Assistance, and Social Security and Labour. The possibility of creating a Higher Labour Council was also discussed. In March 1925, the Workers’ Housing Council was transformed into the *Consejo Superior de Bienestar Social* (Higher Social Welfare Council).^108^ In June, the Higher Public Assistance Council was eliminated and,^109^ in October, the possibility of creating a *Dirección de Subsistencia* (Subsistence Directorate) was discussed.^110^

The constant modifications of the Ministry’s structure indicate that, when it was created, there was a lack of clarity about how to organise the sector. Although Santos Salas designed an organisational structure, it was far from generating consensus. When President Arturo Alessandri resigned in October 1925, Santos Salas also quit as minister in order to run for president. He was succeeded as minister by Pedro Lautaro Ferrer.^111^ The medical community was critical of the work of Santos Salas. For example, Lucio Córdova disapproved of his elimination of the Higher Public Assistance Council, arguing that it reflected a narrow view of the area merely in terms of hospital management.^112^

Between 1924 and 1926, the Ministry carried out its work. In December 1924, it divided the country into nine sanitary zones and, four months later, with the arrival of Salas, tried out a new territorial organisation of health services. In addition, it created the career of public health doctor and,^113^ with Long’s advice, worked on a number of other areas. They included legislative matters and, under Decree Law 602, which came into force on 13 October 1925, a new sanitary code was established. It maintained the General Directorate of Health and the Hygiene Council as central elements in the institutional framework whilst innovating in territorial organisation (see Decree Law 602). Long left his mark on the new code, incorporating international regulation, particularly that of the Philippines and the United States, and the Pan American Sanitary Code. Progress was also achieved in analysing the quality of drinking water. An investigation by the Ministry found that only four out of seventy-five water services were providing ‘reasonably safe’ water. In response, water began to be chlorinated in Santiago, San Antonio, Concepción and Cartagena and investments were made in other cities, such as Valparaíso, with the aim of providing good quality water to 1 250 000 inhabitants (a quarter of the population) by 1927. According to Long, the Ministry’s work had made it possible to provide better milk and generate changes in consumption habits as reflected in higher soap consumption and refrigerator sales as well as an increased tendency to go to the doctor.^114^

During the early years of the Ministry of Hygiene, the General Directorate of Labour was able to consolidate the implementation of labour legislation approved in previous years. In this stage and that which followed with the transition to the Ministry of Social Welfare, enforcement was strengthened,^115^ a task into which female public officials were increasingly incorporated.^116^ In housing, the problem of the price of rents was approached in two ways. The housing tribunals, introduced at the beginning of 1925 by Minister Santos Salas to regulate prices, served as a tool for fostering rent freezes while a Cheap Rooming Law sought to act on prices by encouraging the construction of new rooming houses.^117^

On financial affairs, matters seemed to be moving forward. John D. Long successfully persuaded the commission drafting a new constitution that Article 10, Number 14 should include the assertion that, ‘[i]t is the duty of the state to safeguard public health and the country’s hygienic well-being. A sufficient amount of money must be allocated each year to maintain a national health service’. In addition, the government took out an international loan for CL$7 500 000 to finance the sector.^118^ These were the beginnings of a new state, active in the economic and social spheres.^119^

However, the work of the Ministry was not without difficulties. The Chilean Workers Federation did not agree with workers’ contributions being paid to the government to finance insurance, rather than going to mutual aid societies.^120^ Moreover, the Chilean Medical Society was suspicious of the reorganisation of charity because it implied continuing to use this concept instead of social assistance.^121^ However, perhaps the main problem faced by the Ministry was the stance of some sectors of Congress on health policies.

**Figure 1.**
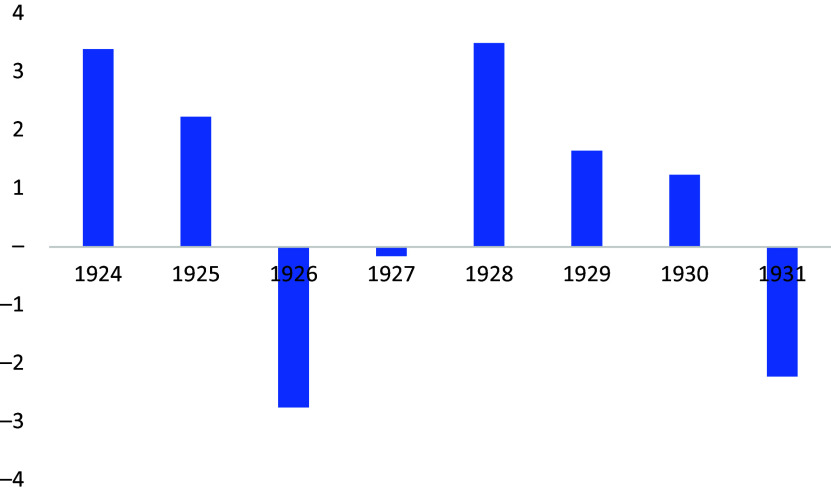
Budget surplus/deficit as percentage of Gross domestic product (GDP). *Source:* José Díaz, Rolf Lüders and Gert Wagner, *La República en cifras. Chile 1810–2010* (Santiago, Ediciones UC, 2016).

This problem became apparent when, in 1926, only three months after President Emiliano Figueroa took office, fiscal finances ran into difficulties (Figure 1), prompting debate about control of public spending.^122^ Attempts to control fiscal spending caused a dispute between Minister of Finance Jorge Silva Somarriva and Minister of Hygiene Lucio Córdova. The Ministry of Finance was reluctant to fund the Ministry of Hygiene’s public health plan. According to *La Nación*, the tension between the two ministries showed that in the government mind, public health does not yet represent more than an ‘item of superfluous expenses’.^123^ In Congress, there were differences between parliamentarians and the executive. The Hygiene and Public Assistance Commission opposed the Ministry of Finance’s idea of reducing the salaries of health personnel.^124^

When the Ministry of Finance rejected a series of appointments of health personnel, which had been approved by the president and the minister of hygiene,^125^ Long resigned. The situation angered the press, which took the view that: ‘He [Long] is leaving out of honesty because his scientific convictions do not allow him to continue “collabourating” any longer in the depopulation of the country’.^126^

The fiscal difficulties did not affect only health workers. Law N° 4.075, approved in July 1926, ordered a reduction in the salaries of public employees in general. Minister Lucio Córdova reminded the Senate that, under the new constitution, public health must be adequately financed but to no avail.^127^ In the following months, debate was not about the budget for the Ministry of Hygiene but its very existence and that of the Ministry of Agriculture. In the face of some proposals along these lines, voices were raised in their defence. For example, according to *El Mercurio*, ‘Both Ministries have worked to the evident advantage of the country and full satisfaction of all the organisations interested in progress in these fields’.^128^ In November, a 20 percent reduction in the Ministry of Hygiene’s budget was mooted as well as the elimination of some jobs as part of a general reduction across the public administration.^129^ Minister Córdova warned Congress that such cuts would be harmful because the Ministry required specialised personnel to head each of its undersecretariats.^130^

Some members of Congress expressed favourable opinions about the Ministry while the press underlined its social role:

The Ministry of Hygiene, Social Security and Labour which, given its purpose, could well be called simply Ministry of Social Medicine (…)

They are, all of them, public services through which the state ensures justice and well-being in the community, to satisfy the duty imposed by modern social conceptions.^131^

The latter part of 1926 brought a political and economic crisis. In addition to the fiscal emergency, Congress was blocking the work of the executive. President Figueroa attempted to alleviate the situation by taking out an overseas loan for US$70 000 000. In November 1926, he also reshuffled his cabinet, but this did not improve the situation. In February 1927, he appointed Ibáñez del Campo as Minister of Interior but, two months later, resigned from the presidency, due to disagreements with Ibáñez del Campo, who had become Chile’s leading political figure.^132^

## The Ministry of Social Welfare

The appointment of Pablo Ramírez as Minister of Finance in February 1927 marked the start of a process of rationalisation and the introduction of a technocratic style of management.^133^ Ramírez focused on implementing Laws N° 4.075 and 4.113, passed in January 1927, which gave broad powers to reorganise finances and administration, and on setting up new institutions such as the Office of the Comptroller General of the Republic and the General Treasury of the Republic.

In March and April, various decrees were issued declaring positions vacant in services reporting to the Ministry of Hygiene. The process began at a meeting on March 11 at which Minister Isaac Hevia, together with the heads of services, decided on the positions to be eliminated.^134^ In the Ministry itself, six positions were declared vacant while, in the General Directorate of Health, 75 were eliminated in March and 15 in April. It was also decided not to fill another five positions that had previously become vacant. In the Directorate of Labour, seventeen positions were suppressed, and it was decided not to fill another two. In April, the twenty positions of regional inspector were eliminated. The General Directorate of Social Assistance, the Higher Welfare Council, the Social Illnesses Dispensary and the Valparaíso Dispensary discarded ten, seven, twenty-five and five positions, respectively, while the Health Directorate abolished its presidents and inspectors. A reduction in personnel was not the only objective of these decrees; they were also used to put new people in charge of the different services. In the case of the Ministry, the positions of undersecretary of hygiene and undersecretary of social security and labour were declared vacant, as well as those of the heads of the Social Assistance, International, Libraries and Publications, and Cooperation, Mutuality and Welfare sections. The positions of director of the General Directorate of Health, the General Directorate of Labour, the Social Illnesses Dispensary and the General Directorate of Social Assistance were also eliminated. As a result, prominent officials such as Óscar Donoso, Moisés Poblete, Lucas Sierra, Alfredo Weber, Julio Bustos and Lucio Córdova lost their jobs.^135^

However, a change of direction soon followed. In an interview with *El Mercurio*, Undersecretary Álvarez indicated that the Ministry was looking at replacing the existing councils to transform them into advisory bodies, based on a logic of functional representation.^136^ Similarly, when opening Congress, Vice President Carlos Ibáñez del Campo stated that:

Until recently, social assistance by the state has led a languid life, [due to] the scarcity of resources, on the one hand, and the lack of precise orientation, on the other… Today, intensive work is being done to organise in a well-defined way so that it [social assistance] can be enjoyed not only by the inhabitants of the big cities but especially by the poor and needy of small towns and the countryside.^137^

In August, a decision was taken to transfer some of the Ministry’s enforcement functions to regional and provincial governors and mayors in order to decongest the central level and transform it into ‘research labouratories, institutes that will study in practice the best adaptation of modern theories for their efficient application to the citizens who will receive their benefits’.^138^

At the end of 1927, the Ibáñez government reorganised ministries. The Ministry of Agriculture was eliminated and Hygiene was transformed into the Ministry of Social Welfare (Decree with Force of Law 7.912 of 1927). It was designed through Decree with Force of Law (DFL) 2.024, also issued in 1927. The new name implied a shift in the focus of the Ministry which had, until then, covered the spheres of health, labour and housing, with priority given to health. The notion of social welfare implied moving towards a new approach that included other aspects relevant to the well-being of the population, such as water and pensions. As a result, the *Dirección General de Hidráulica* (General Directorate of Hydraulics) and the public and municipal employees’ compensation funds were incorporated into the Ministry. This was consistent with the logic of the reform being implemented, which sought to rationalise the number of services. Indeed, the second point of DFL 2.024 stated that the Ministry’s effectiveness called for ‘the simplification of current services, eliminating those that are superfluous and reducing those that are indispensable to their proper size so that the service is simple, effective and economic’. Moreover, the Ministry of Social Welfare was inaugurated with clarity regarding the aspects it should address.

On housing, which had until then only been regulated in relation to mid-income workers and employees, DFL 2.024 stated that it was also necessary to expand coverage to other actors who ‘live without the most elementary hygienic conditions, alongside the opulence of the employers’ housing’. It also noted the lack of supervision of construction practices in which ‘scientific principles are dispensed with in favour of excessive profit or mere whim’. In the case of health, which the decree considered ‘complex, burdensome and ineffective because of a lack of cooperation on the part of the administrative authorities of the Republic’, it proposed giving these authorities the powers to implement existing legislation. On public assistance, the aim was to ‘unify the social action of this service in order to reach the largest number of our fellow citizens and take proper advantage of the huge expenses currently incurred by the State’. Finally, the decree noted the challenge of implementing social security laws while, as regards labour inspection services, it urged that they should have ‘the characteristics of justice and equity, which allow capital to function confidently and ensure that workers’ rights and institutional progress will be duly respected with the support of the government and the cooperation of the employer, thus avoiding the demands of the former and the egoism of the latter’. A new sphere of action was added to these functions: the management of water and sewage collection services.

As a result, the Ministry comprised the minister’s office, the undersecretariat, the Social Security Department, the Rooming Construction Technical Department and the Social Assistance Technical Department. In addition, the following services reported to it: the General Directorate of Health, the General Labour Inspectorate and the General Directorate of Hydraulics. Its 1928 budget also included the Santiago Province’s Directorate of Sewers and Paving and the Valparaíso Drinking Water Company.^139^

When opening Congress in 1928, Ibáñez laid out an ambitious plan for the new Ministry. On health, he indicated that it would become a ‘more economical and efficient organisation’, adding that he hoped to achieve ‘control of all the physical ills that afflict our population’, implement a ‘salvation campaign’ to protect the country’s children and mount an attack on tuberculosis.^140^

Ibáñez’s arrival to power had changed the fortunes of the social ministry created in 1924. It went from being under question to being reorganised in a way that enabled it to expand its field of action. This is reflected in Table 3. In 1927, the Ministry of Hygiene captured 2.6 percent of the fiscal budget, a figure that rose by one percentage point in the following three years.

**Table 3.** Expenditure of ministries, 1925–1931, in Chilean pesos

Ministry	1925	1926	1927	1928	1929	1930	1931
Interior	81 542 296	140 816 135	133 651 255	120 497 743	133 851 343	151 719 740	150 729 740
Foreign Relations and Religion	1 420 641	10 670 276	9 266 889	11 727 220	17 607 480	17 366 600	10 854 000
Finance	72 987 151	289 909 979	298 290 274	386 204 068	412 627 454	434 221 209	517 654 559
Education	85 529 645	140 593 637	141 887 605	130 003 072	145 029 554	164 244 388	131 498 112
Justice	20 588 752	27 585 553	28 669 774	23 550 856	26 236 352	30 442 835	25 668 669
War	80 602 427	113 489 605	115 228 669	106 160 000	112 724 585	121 234 963	90 522 194
Navy	45 997 871	94 471 297	97 053 786	102 958 424	114 915 815	119 769 062	89 668 282
Agriculture and Industry	12 070 280	15 690 162	14 471 445				
Public Works and Roads	47 287 604	92 507 319	126 579 267				
Hygiene, Assistance and Social Security	21 879 355	30 992 140	26 355 877				
Lands and Colonisation	1 715 255	2 098 436	1 980 925				
Economic Development				17 800 000	55 895 220	62 274 055	
Social Welfare				33 775 000	40 662 230	41 642 160	7 622 900
Agriculture							5 458 784
General Directorate of Public Works							1 928 600
Southern Property							4 349 000
Total	471 621 277	958 824 539	993 435 766	932 676 383	1 059 550 033	1 142 915 012	1 035 954 840

*Source:* Chile, *Ley de presupuestos de gastos generales de la administración pública* (Santiago: Imprenta Nacional, 1925–1931).

However, its increased share of fiscal resources was accompanied by constant debate about the form the Ministry should take. The initial health-labour-housing triad went on to take different configurations. In 1929, the *Inspección del Trabajo* (Labour Inspection Service) was given housing responsibilities and the *Junta Central de Beneficencia* (Central Charity Board) was added to the Ministry.^141^ A bill to create a Bacteriological Institute was also presented.^142^ A year later, the Central Charity Board was transformed into the *Dirección General de Beneficencia y Asistencia Social* (General Directorate of Charity and Social Assistance) while the Valparaíso Drinking Water Company ceased to be part of the Ministry and both the General Directorate of Hydraulics and the Santiago Province’s Directorate of Sewers and Paving were transferred to the Interior Ministry.^143^ In addition, the creation of an Inspectorate of Cooperatives as part of the Ministry was mooted.^144^ In 1931, it was decided to transfer the Rooming Construction Technical Department to the Ministry of Economic Development’s General Directorate of Public Works.^145^

As well as being part of an effort to devise a better way of organising the public administration, these changes sought to give consistency to the idea of welfare. While the ministry was the Ministry of Hygiene, the health sector accounted for most of its spending but, from 1928, health began to lose ground relative to public works and sewage collection (Table 4). In line with this, Ibáñez told Congress in 1930 that the main concerns of the Ministry of Social Welfare were the harmonisation of capital and labour, social security and health.^146^

**Table 4.** Distribution of the budget of the Ministries of Hygiene and Welfare, by sector, in percentages

Sector	1925	1926	1927	1928	1929	1930	1931
Health	95.03	76.53	90.36	67.55	61.70	63.37	42.72
Labour	2.12	8.53	3.67	1.13	0.26	0.25	46.49
Housing	1.83	10.86	0.66	1.13	0.94	1.24	5.60
Public works and sewers				30.19	30.39	27.79	
Welfare, childhood, physical education	0.66						

*Source:* Compiled by authors based on Chile, *Ley de presupuestos de gastos generales de la administración pública* (Santiago, Imprenta Nacional, 1925–1931).

By 1930, voices began to be raised urging the need to reform the ministry and create one exclusively for public health.^147^ The then minister, Humberto Arce, argued against eliminating a ministry focused on ‘social problems because, with great reason, these problems are of the highest political importance in the true sense of the word’. Similarly, *El Mercurio* considered that the ministry was the target of ‘premature criticism’ and should be given time to show the expected results.^148^

Indeed, some achievements were already becoming apparent. According to the government, Chile had achieved the lowest mortality rate of its history in 1928.^149^ Hospital capacity was another area that was showing progress. In 1928, Ibáñez announced a plan to expand it and,^150^ three years later, the results were clear. Between 1927 and 1931, the number of hospitals increased by 27.1 percent, the number of people attended by 25.1 percent, doctors by 27.8 percent and beds by 35.1 percent (Table 5). However, the process was not without its problems. For example, in the Senate, Carlos Villarroel, a liberal, cited a report issued by the Ministry of Social Welfare itself in 1930, indicating that, out of 100 hospitals, eighty-five did not have labouratories, seventy-seven lacked X-ray equipment, fifty-three did not have a maternity service and twenty-five did not have an operating theatre.^151^

**Table 5.** Hospitals, personnel, patients and beds, 1925–1931

	1925	1926	1927	1928	1929	1930	1931
Hospitals	119	117	118	125	141	145	150
N° of patients attended	149 950	153 903	165 442	171 449	197 616	206 183	207 059
N° of doctors	649	631	632	886	946	963	808
Total personnel	5,310	5,249	5,579	6,656	7,579	7,652	7,798
N° of beds	12 614	12 656	11 895	13 398	14 516	15 697	16 076

* Doctors and other personnel include both external and internal personnel.

** Personnel initially corresponds to doctors, trainees and other personnel to whom dentists, nurses and chemists are subsequently added.

*** Beds includes both free and paid beds.

*Source:* Oficina Central de Estadística, *op. cit.* (Table 2).

The feeling of prosperity that had been a feature of the presidency of Carlos Ibáñez del Campo dissipated in 1931 as Chile began to suffer the impact of the Great Depression.^152^ Initially, the government attempted to address the situation through fiscal tightening, reducing public spending under the 1931 budget to 90.6 percent of its level in 1930. The case of the Ministry of Social Welfare was particularly dramatic: its budget was cut to a mere 18.3 percent of its level in 1930 (Table 3). However, these measures did not suffice: by the middle of the year, social protest had become unsustainable and, on 26 July 1931, Ibáñez was forced to resign. In 1932, the Ministry of Social Welfare was divided into two new ministries: Health and Labour, with the latter taking responsibility for housing. This marked the end of the first phase of the country’s social institutions in which their development was facilitated by the political crisis but limited by economic restrictions. From then on, the ministerial structure would undergo further changes, opening the way to new and more specialised ministries with more specific areas of intervention.

## Conclusions

Through an analysis of the creation of Chile’s Ministry of Hygiene, Assistance and Social Security in the wake of the revolution of September 1924, this paper has sought to contribute to debate about the institutionalisation of health ministries in Latin America. The second section has shown that, from the 1880s, most of the region’s countries created departments for health matters. This process was common to the different countries and reflected a need to address health problems related to economic modernisation and urbanisation.

However, when examining the creation of ministerial-level bodies, differences appear, revealing two distinct paths of institutional development. The first, seen in a group of precursor countries, principally in the Caribbean and Central America, reflects the fact that, for various reasons, they were influenced (and, in the case of the Dominican Republic, occupied) by the United States which, from the 1880s, promoted the adoption of health measures in the region. The second road is characterised by the creation of ministries in the wake of the emergence of new political and military figures or populist leaders, who sought to establish a new type of state. This occurred mostly from 1930, with the exception of Chile and Ecuador (1924 and 1925). Why were ministries created under this scheme?

The analysis of the Chilean case presented here provides some answers. It shows that ideas about the creation of a health ministry arose in the 1920s in response to growing public demand for a greater state presence in this field as well as on labour issues. However, these came up against a political context dominated by parliamentary obstruction of Alessandri’s agenda. Ideas of this type were at odds with the efforts made, at different times, to control the size of the public administration. The so-called September Revolution and its ideas for social reform produced precisely the conditions for the creation of a new ministry as explained in 1926 by *El Mercurio*:

The purpose of creating these Ministries existed. But, since our Congress was incapable of carrying out any positive work and spent several years engaged in hindering the government and bringing down ministries, it never managed to dispatch a bill on the subject.

The revolutionary period was used to materialise many national aspirations, twenty or thirty years old, that parliamentary sterility had held back and were unanimously proclaimed as just.^153^

The new political context provided an opportunity to modify the structure of ministries and created a new space for questions of a social nature. However, this new ministry’s institutional development was also affected by long-term administrative trends, with their roots in the country’s parliamentary period. Initially, the ministry was not conceived as a unit with a clear purpose but, rather, as a response to the grouping of health with two other areas of social intervention in Chile during the early decades of the twentieth century: labour and housing. Why were these sectors grouped together under a single ministry? Was this the result of a conception of welfare that called for a combination of actions in the different fields? The answer appears to be that this was not the case. The creation of a ministry of health and of a ministry of labour were proposed in different contexts. The former was proposed by the medical community as its influence in public debate grew. By contrast, the idea of a ministry exclusively for labour matters arose as a bid to establish mechanisms for mediation between capital and labour. The grouping of these three areas within a single ministry appears to have been mainly a result of the implementation, as from the 1890s, of a form of organisation of the public administration that consisted in the merger and centralisation of agencies into new bodies. This represented a bid to contain fiscal spending in the face of fiscal crises that, from the 1910s, became recurrent. Indeed, as shown here, the budget deficit was an important concern to the point of jeopardising the ministerial project.

A new stage began when President Ibáñez took office in 1927. The creation of the Ministry of Social Welfare sought not only to solve administrative problems, but also to give the ministry a new focus, incorporating other responsibilities, such as access to drinking water and pensions, that, like health, labour and housing, affect people’s quality of life. However, this effort was short-lived since, for example, sewage collection services were returned to the Ministry of Interior. Finally, the coexistence of health, labour and housing was maintained until, in 1932, the ministry was split into the Ministry of Health and the Ministry of Labour. Paradoxically, housing which, with its obvious impact on quality of life, had a clear relationship with health, ended up in the Ministry of Labour.

Future research should explore, from a comparative standpoint, the usefulness of the two trajectories identified here in explaining the creation of health ministries in Latin America. Similarly, for countries that follow the path of institutional development after breakdowns and/or the emergence of populist projects, the findings of the Chilean case presented here may serve as a guide. In addition, this research may contribute to a deeper understanding of the emergence and institutionalisation of the social medicine approach in Latin America, looking beyond the role of doctors, on which accounts generally focus, and incorporating the role of political changes and, especially, the rise of progressive members of the armed forces.

## Footnotes

1

Marcos Cueto and Steve Palmer , Medicine and Public Health in Latin America: a History (New York: Cambridge University Press, 2015).

2

Mariola Espinosa , ‘Globalizing the History of Disease, Medicine, and Public Health in Latin America’, Isis, 104, 4 (2013), 798–806 24783496 10.1086/674946.

3
*Ibid.*

4

Marcos Cueto , ‘The Cycles of Eradication: The Rockefeller Foundation and Latin American Public Health: 1918–1940’, in Paul J. Weindling . (ed.), International Health Organizations and Movements, 1918–1939 (Cambridge: Cambridge University Press, 1995), 222–43; Marcos Cueto , The Value of Health. A History of the Pan American Health Organization (Washington, D.C.: PAHO, 2006); Cueto *et al*., *op. cit.* (note 1).

5

Anne-Emanuelle Birn , ‘Public Health and Medicine in Latin America’, in M. Jackson (ed.), The Oxford Handbook of the History of Medicine (New York: Oxford University Press, 2011), 243–64.

6

Juan C. García , ‘La medicina estatal en América Latina (1880–1930)’, Revista Cubana de Salud Pública, 42, 1 (2016), 143–75.

7

Patricio Márquez and Daniel Joly , ‘A Historical Overview of the Ministries of Public Health and the Medical Programs of the Social Security Systems in Latin America’, Journal of Public Health Policy, 7, 3 (1986), 378–943533995 ; Anne-Emaneulle Birn and Laura Nervi , ‘Political Roots of the Struggle for Health Justice in Latin America’, The Lancet, 385, 9974 (2015), 1174–510.1016/S0140-6736(14)61844-425845782.

8 Márquez *et al*., *op. cit.* (note 7).

9

José Portillo , ‘Historia de la medicina estatal en Uruguay (1724–1930)’, Revista Médica de Uruguay, 11 (1995), 5–18 ; Marcelo Setaro, ‘Vigilar y cuidar el bien común: el rol de rectoría del sistema de salud desde una perspectiva comparativa’ (Master’s thesis, Universidad de la República, 2004); Pablo Camus and Felipe Zúñiga , ‘La Salud Pública en la Historia de Chile’, Anales Chilenos de la Historia de la Medicina, 17, 2 (2007), 155–75; Federico Tobar , ‘Breve historia del sistema argentino de salud’, in Oscar Garay (ed.), Responsabilidad profesional de los médicos. Ética, bioética y jurídica. Civil y penal (Buenos Aires: La Ley, 2012).

10 Cueto, *op. cit.* (note 4); Xinia Bustamante (ed.), 100 años de salud en Costa Rica: Siglo XX (San José: PAHO, 2003); René Salinas , ‘Salud, ideología y desarrollo social en Chile: 1830–1950’, Cuadernos de Historia, 3 (1983), 99–126 ; Gonzalo Saraví (ed.), De la pobreza a la exclusión. Continuidades y rupturas de la cuestión social en América Latina (Buenos Aires, CIESAS/Prometeo, 2006); María A. Illanes , En el nombre del Pueblo, del Estado y de la Ciencia. Historia social de la salud pública, Chile, 1880–1973: hacia una historia social del Siglo XX (Santiago, Colectivo de Atención Primaria, 1993); García, *op. cit.* (note 6).

11 García, *op. cit.* (note 6).

12
*Ibid.*, 145.

13 Márquez *et al*., *op. cit.* (note 7).

14 García, *op. cit.* (note 6).

15

Eric D. Carter , ‘Social Medicine and International Expert Networks in Latin America, 1930–1945’, Global Public Health, 14, 6-7 (2019), 791–802 29297771 10.1080/17441692.2017.1418902.

16

Gregorio Delgado , ‘Desarrollo histórico de la Salud Pública en Cuba’, Revista Cubana de Salud Pública, 24, 2 (1998), 110–8; García, *op. cit.* (note 6); María T. Gutiérrez , ‘Proceso de institucionalización de la higiene: Estado, salubridad e higienismo en Colombia en la primera mitad del siglo XX’, Estudios Socio-Jurídicos, 12, 1 (2010), 73–97 .

17

Roberto Hernández , ‘La atención médica en Cuba hasta 1958’, Journal of Inter-American Studies, 11, 4 (1969), 533–57; Iveth Rubán Rodríguez , ‘Un acercamiento a la historia de la salud pública en el Bayamo Republicano (1902–1928)’, Revista de Ciencias Sociales, 162, 4 (2018), 169–83; Everardo Duarte Nunes , ‘Sobre a história da saúde pública: idéias e autores’, Ciência & Saúde Coletiva, 5, 2 (2000), 251–64; Gilson Carvalho , ‘A saúde pública no Brasil’, Estudos Avançados, 27, 78 (2013), 7–26 ; Cueto *et al*., *op. cit.* (note 1); Carolina Biernat, ‘Continuidades y rupturas en el proceso de centralización de la administración sanitaria argentina (1880–1945)’, Trabajos y Comunicaciones, 44, e021 (2016), available at https://www.trabajosycomunicaciones.fahce.unlp.edu.ar/article/view/TyCe021.

18 Illanes, *op. cit.* (note 3); William Sater , ‘The Politics of Public Health: Smallpox in Chile’, Journal of Latin American Studies, 35, 3 (2003), 513–43.

19 Portillo, *op. cit.* (note 9); Setaro, *op. cit.* (note 9).

20

Gregorio Delgado , Eduardo Estrella and Judith Navarro , ‘El Código Sanitario Panamericano hacia una política de salud continental’, Revista Panamericana de Salud Pública, 6, 5 (1999), 350–61.

21 Emilio Quevedo, ‘¿Políticas de salud o políticas insalubres? De la higiene a la salud pública en Colombia en la primera mitad del siglo XX’, *Biomédica*, 16 (1996), 345–60; Cueto, *op. cit.* (note 4).

22 Birn, *op. cit.* (note 5); García, *op. cit.* (note 6).

23 Cueto *et al*., *op. cit.* (note 1); Julian Eckl, ‘The Power of Private Foundations: Rockefeller and Gates in the Struggle against Malaria’, *Global Social Policy*, 14, 1 (2014), 91–116; Quevedo, *op. cit.* (note 21); Armando Solórzano , ‘La influencia de la Fundación Rockefeller en la conformación de la profesión médica mexicana, 1921–1949’, Revista Mexicana de Sociología, 58, 1 (1996), 173–203 .

24 García, *op. cit.* (note 6).

25 Illanes, *op. cit.* (note 10).

26 García, *op. cit.* (note 6); Illanes, *op. cit.* (note 10); Portillo, *op. cit.* (note 9); Susana Belmartino , La atención médica argentina en el siglo XX: instituciones y procesos (Buenos Aires: Siglo Veintiuno Editores, 2005); Ana P. Malavassi , ‘El encuentro de la Fundación Rockefeller con América Central, 1914–1921’, Diálogos: Revista Electrónica de Historia, 7, 1 (2006), 115–49.

27 Delgado, *op. cit.* (note 20); Néstor Marimón and Evelyn Martínez , ‘Protección de las conquistas de la salud pública cubana ante la crisis financiera y económica mundial’, Revista Cubana de Salud Pública, 36, 4 (2010), 337–48; Rubán Rodríguez, *op. cit.* (note 17).

28

Leobardo Ruiz , Carlos Viesca , Fernando Martínez , Guillermo Fajardo , Carlos Castañeda , Azucena Galindo , Xóchitl Martínez , Mariblanca Ramos and Gabino Sánchez , ‘Antecedentes y evolución de la salubridad pública en el México independiente’, in Fernando Gutiérrez (coord.), Secretaría de Salud: la salud en la Constitución mexicana (Ciudad de México, INEHRM, 2018), 23–72 .

29

Ernesto Aréchiga , ‘«Dictadura sanitaria», educación y propaganda higiénica en el México Revolucionario, 1917-1934’, Dynamis: Acta hispanica ad medicinae scientiarumque historiam illustrandam, 25 (2005), 117–4317345657 ; Ana C. Rodríguez and Martha Rodríguez , ‘History of Public Health in Mexico: 19th and 20th Centuries’, História, Ciências, Saúde – Manguinhos, 5, 2 (1998), 293–310 16646153 10.1590/s0104-59701998000200002.

30 García, *op. cit.* (note 6).

31

Pan American Health Organization, The Pan American Sanitary Code. Toward a Hemispheric Health Policy (Washington, DC: PAHO, 1999).

32 García, *op. cit.* (note 6).

33 Cueto *et al*., *op. cit.* (note 1).

34 García, *op. cit.* (note 6); Cueto, *op. cit.* (note 4), Carter, *op. cit.* (note 15).

35 García, *op. cit.* (note 6).

36 Marquez *et al*., *op. cit.* (note 7).

37

Peter Smith and Thomas Skidmore , Modern Latin America (New York: Oxford University Press, 1980); Víctor Bulmes-Thomas , La historia económica de América Latina desde la Independencia (Ciudad de México: Fondo de Cultura Económica, 2017).

38

Rodolfo Pastor , Historia mínima de Centroamérica (Ciudad de México: El Colegio de México: 2011).

39 Bulmes-Thomas, *op. cit.* (note 37).

40

Thomas Schoonover , ‘A United States Dilemma: Economic Opportunity and Anti-Americanism in El Salvador, 1901–1911’, Pacific Historical Review, 58, 4 (1989), 403–28; Philip F. Dur , ‘US Diplomacy and the Salvadorean Revolution of 1931’, Journal of Latin American Studies, 30, 1 (1998), 95–119 ; Héctor Lindo-Fuentes , ‘El Salvador vs. Imperialismo Yanqui, 1912–1914’, Journal of Latin American Studies, 52, 3 (2020), 495–519 .

41 Dur, *op. cit.* (note 40).

42 Cueto, *op. cit.* (note 4).

43

Joseph L. Arbena , ‘Colombian Reactions to the Independence of Panama, 1903–1904’, The Americas, 33, 1 (1976), 130–48.

44

Patricia Cardona , ‘Panamá: el istmo de la discordia. Documentos relativos a la separación de Panamá y a la normalización de las relaciones entre Estados Unidos y Colombia’, Araucaria. Revista Iberoamericana de Filosofía, Política y Humanidades, 17, 33 (2015), 281–305 ; Xavier Duran and Marcelo Bucheli , ‘Holding Up the Empire: Colombia, American Oil Interests, and the 1921 Urrutia–Thomson Treaty’, The Journal of Economic History, 77, 1 (2017), 251–84.

45

Paul Drake , The Money Doctor in the Andes. The Kemmerer Missions, 1923–1933 (Durham: Duke University Press, 1989).

46 Quevedo, *op. cit.* (note 21).

47 Drake, *op. cit.* (note 45), Quevedo, *op. cit.* (note 21); Gutiérrez, *op. cit.* (note 16).

48 Malavassi, *op. cit.* (note 26).

49 According to Drake, the September Revolution influenced the July experience in Ecuador. Drake, *op. cit.* (note 45).

50 Drake, *op. cit.* (note 45); Juan J. Paz y Miño, *La Revolución Juliana: nación, ejército y bancocracia* (Quito: Editorial Abya Yala, 2000).

51 García, *op. cit.* (note 6).

52

A. Kim Clark , ‘New Arenas of State Action in Highland Ecuador: Public Health and State Formation, c. 1925–1950’, in Christopher Kupra and David Nugent (eds.), State Theory and Andean Politics. New Approaches to the Study of Rule (Philadelphia, PA: University of Pennsylvania Press, 2015).

53

Oscar Mac-Clure , En los orígenes de las políticas sociales en Chile 1850–1879 (Santiago: Ediciones Universidad Alberto Hurtado, 2012).

54 Illanes, *op. cit.* (note 10).

55

Diego Barría , ‘Bureaucratization in a Changing Society: Administrative Reforms in Late Nineteenth Century Chile’, International Journal of Public Administration, 41, 7 (2018), 562–72.

56

Sergio Grez , ‘La trayectoria histórica del mutualismo en Chile (1853–1990): apuntes para su estudio’, Mapocho: Revista de Humanidades y Ciencias Sociales, 35 (1994), 293–315 .

57

Peter DeShazo , Trabajadores urbanos y sindicatos en Chile: 1902–1927 (Santiago: Centro de Investigaciones Diego Barros Arana, 2007).

58
*Ibid.*

59

Manuel Bastías , ‘Intervención del Estado y derechos sociales. Transformaciones en el pensamiento jurídico chileno en la era de la cuestión social, 1880–1925’, Historia, 48, 1 (2015), 11–42 .

60

Juan C. Yáñez , Estado, consenso y crisis social. El espacio público en Chile 1900–1920 (Santiago: Centro de Investigaciones Diego Barros Arana, 2003).

61 Illanes, *op. cit.* (note 10); Barría, *op. cit.* (note 55).

62 Del Río graduated as a doctor in 1890 and, between 1891 and 1894, specialized in hygiene in Europe. He played a prominent role in the establishment of the Santiago Public Hospital in 1911 and also chaired the V American Health Conference that year. Ferrer graduated in 1905 and, in 1907, was appointed health inspector. Two years later, he took charge of the Hygiene Section of the Ministry of Interior. He drew up the 1920 beneficence regulations and was one of the founders of the Chilean Red Cross. Lucio Córdova graduated in 1898, after which he specialized in France. In 1912, he joined the University of Chile as an academic and, in 1921, was appointed director of the Salvador Hospital. Ricardo Cruz-Coke , Historia de la medicina chilena (Santiago, Editorial Andrés Bello, 1995).

63

Federico Puga Borne , ‘Antecedentes de la organización del servicio de Hijiene Pública en Chile’, Revista chilena de Hijiene, I, 1 (August 1894), 6 .

64

Alejandro del Río , ‘Tercera Memoria del Instituto de Hijiene de Santiago’, Revista chilena de Hijiene II, 7 (May 1896), 229–30; del Río, ‘Cuarta Memoria del Instituto de Hijiene de Santiago’, Revista chilena de Hijiene, III, 11 (May 1897), 147–9; del Río, ‘Quinta Memoria del Instituto de Hijiene correspondiente al año de 1897’, Revista chilena de Hijiene, VI, 15 (May 1898), 256 .

65 Ministerio del Interior, *Ley 1079bis sobre Inspectores Sanitarios* (28 septiembre 1898): https://www.bcn.cl/leychile/navegar?idNorma=22396.

66

Ministerio del Interior, Memoria del Ministro del Interior presentada al Congreso Nacional en 1900 (Santiago: Imprenta Nacional, 1900), 31 .

67

Carlos Molina , Institucionalidad sanitaria chilena, 1889–1989 (Santiago: LOM, 2010).

68

Ministerio del Interior, Memoria del Ministro del Interior presentada al Congreso Nacional en 1907 (Santiago: Imprenta Nacional, 1907), 78 .

69

Oficina Central de Estad í stica, Anuario Estadístico de la República de Chile (Santiago: Oficina Central de Estadística, 1909).

70

Juan C. Yáñez , La Intervención Social en Chile, 1907–1932 (Santiago: RIL Editores, 2008); Daniel Ahumada and Alejandro Salas , ‘La participación de la Oficina del Trabajo en la configuración del sistema de previsión social chileno, 1909–1925’, Revista Historia y Geografía, 39, 2 (2008), 73–97 .

71

Rodrigo Hidalgo , La vivienda social en Chile y la construcción del espacio urbano en el Santiago del siglo XX (Santiago: Centro de Investigaciones Diego Barrios Arana, 2005).

72 Yáñez, *op. cit.* (note 64).

73

Patrick Barr-Melej , Reforming Chile: Cultural Politics, Nationalism, and the Rise of the Middle Class (Chapel Hill: University of North Carolina Press, 2001).

74

Ministerio del Interior, Memoria del Ministro del Interior presentada al Congreso Nacional en 1911 (Santiago: Imprenta Nacional, 1911), 22–3.

75

Ministerio del Interior, Memoria del Ministro del Interior presentada al Congreso Nacional en 1917 (Santiago: Imprenta Nacional, 1917), 22 .

76

Germán Valenzuela , ‘Memoria presentada por el Presidente de la Sociedad Médica’, Revista Médica de Chile, XLVII, 9 (September 1919), 545 .

77

Cristina Moyano and Javier Rivas , ‘De agendas, saber experto, redes informales y formas de intervención sociopolítica. Los casos de la administración del Estado y la salud pública en la década de 1920’, in Mauricio Olavarría (ed.), Historia y políticas públicas. Formulación de políticas públicas en la vieja democracia chilena (Santiago: RIL, 2022), 114–6; Ricardo Dávila Boza , ‘Situación sanitaria del país’, Revista Chilena de Higiene, XXI, 93 (1919), 119 .

78 Cueto *et al*., *op. Cit.* (note 1).

79 Illanes, *op. cit.* (note 10).

80 Cruz-Coke, *op. cit.* (note 62).

81 N/A, ‘Editorial’, *Revista Chilena de Higiene*, XXV (1919), 5; N/A, ‘Reveladora lección’, *Revista Chilena de Higiene*, XXVII (1921), 5.

82 Illanes, *op. cit.* (note 10).

83 Yáñez, *op. cit.* (note 64), 252–3; Illanes, *op. cit.* (note 10), 185.

84 Hidalgo, *op. cit.* (note 65).

85

Sofía Correa , Consuelo Figueroa , Alfredo Jocelyn-Holt , Claudio Rolle and Manuel Vicuña , Historia del siglo XX chileno: balance paradojal (Santiago: Editorial Sudamericana, 2001).

86

Fernando Pinto Lagarrigue , Balmaceda y los Gobiernos seudo-parlamentarios (Santiago: Editorial Andrés Bello, 1991).

87

Julio Heise , Historia de Chile: El período parlamentario, 1861–1925, vol. I (Santiago: Editorial Andrés Bello, 1974).

88 Correa *et al*., *op. cit.* (note 75).

89

Ministerio del Interior, Memoria del Ministro del Interior presentada al Congreso Nacional en 1921 (Santiago: Imprenta Nacional, 1921), 49 .

90

Germán Valenzuela , ‘Encuesta sobre el Proyecto de Ley de Organización de la Beneficiencia Pública’, Revista Médica de Chile, XLVII, 6 (June 1919), 312–9.

91
Dr. Espina , ‘Creación de un Ministerio de Sanidad y Asistencia Social’, Revista Médica de Chile, XLIX, 1 (January 1921), 105–6; Dr. Lois , ‘Proyecto de creación del Ministerio de Sanidad y Asistencia Social’, Revista Médica de Chile, L, 7–8 (July-August 1922), 436–8); Federico Puga Borne , ‘La Dirección General de Sanidad I’, Revista Médica de Chile, L, 9–10 (September-October 1922), 560–78; Puga Borne , ‘La Dirección General de Sanidad II’, Revista Médica de Chile, L, 11–12 (November-December 1922), 676–83; Puga Borne , ‘La Dirección General de Sanidad III’, Revista Médica de Chile, LI, 1–2 (January-February 1923), 126–38; Puga Borne , ‘La Dirección General de Sanidad IV’, Revista Médica de Chile, LI, 3–4 (March-April 1923), 239–48.

92 ‘La aplicación del Código Long’, *La Nación*, 11 de julio de 1926.

93 Pinto Lagarrigue, *op. cit.* (note 76).

94

Harry Scott , Pensando el Chile nuevo. Las ideas e la Revolución de los Tenientes y el primer gobierno de Ibáñez, 1924–1930 (Santiago: Centro de Estudios Bicentenario, 2009).

95
*Ibid.*

96 Quoted in Scott, *op. cit.* (note 80), 58.

97 ‘Ministerio de Higiene y la previsión social’, *El Mercurio*, 24 de septiembre de 1924.

98

María A. Illanes , Cuerpo y sangre de la política: la construcción histórica de las visitadoras sociales Chile, 1887-1940 (Santiago: LOM, 2006), 261 .

99 Quoted in Illanes, *op. cit.* (note 10), 207.

100
*Ibid.*, 209.

101 ‘Higiene, asistencia, previsión social’, *El Mercurio*, 25 de octubre de 1924.

102 García, *op. cit.* (note 6).

103 ‘Ayer asumió el cargo de asesor técnico del Ministerio de Higiene el Doctor Long’, *La Nación*, 14 de julio de 1925.

104 N/A, ‘Crónica: el asesor técnico del Ministerio de Higiene y Asistencia Social, Dr. John D. Long, en la Sociedad Médica’, *Revista Médica de Chile* LIII: 10 (October 1925), 506–10.

105 ‘Creación del Ministerio de Higiene, Trabajo asistencia y previsión social’, *El Mercurio*, 16 de octubre de 1924.

106 Yáñez, *op. cit.* (note 64).

107 Scott, *op. cit.* (note 80).

108 Illanes, *op. cit.* (note 10), 214–8; Yáñez, *op. cit.* (note 64), 256–7; Scott, *op. cit.* (note 80), 67–8; ‘Se creará un Consejo Superior del Trabajo’, *El Mercurio*, 7 de febrero de 1925.

109 ‘Es decretada ayer la disolución del Consejo Superior de Asistencia Social’, *El Mercurio*, 21 de junio de 1925.

110 ‘El problema de las subsistencias y el proyecto del ministerio de higiene’, *El Mercurio*, 9 de octubre de 1925.

111 ‘Renuncia el ministro de higiene Dr. José S. Salas’, *El Mercurio*, 11 de octubre de 1925.

112 ‘La labour desarrollada por el Ministerio de Higiene’, *El Mercurio*, 15 de octubre de 1925.

113 Illanes, *op. cit.* (note 10), 213–9.

114

John D. Long , ‘Reorganization of the National Health Service of Chile’, Public Health Reports, 42, 24 (1927), 1617–2319315091 .

115 Yáñez, *op. cit.* (note 64).

116

Diego Barría , Manuel Llorca-Jaña and Nathaly Sepúlveda , ‘The Incorporation of Women into the Public Sector in Chile, 1860–1930: from Rejection to Encouragement’, Management & Organizational History, 16, 2 (2001), 133–55.

117 Hidalgo, *op. cit.* (note 65).

118 Ministerio del Interior, *Actas oficiales de las sesiones celebradas por la Comisión y Sub-comisiones encargadas del estudio del Proyecto de Nueva Constitución* (Santiago: Imprenta Universitaria Será Gobierno de Chile, Ley de Presupuestos de Gastos Generales de la Administración Pública (Santiago: Imprenta Nacional, 1925); Long, *op. cit.* (note 99).

119

Adolfo Ibáñez , Herido en el ala. Estado, oligarquías y subdesarrollo. Chile 1924-1960 (Santiago: Editorial Biblioteca Americana, 2003); Rodrigo Henríquez , Estado Sólido. Políticas y politización en la construcción estatal. Chile, 1920–1950 (Santiago: Ediciones UC, 2014).

120 Illanes, *op. cit.* (note 10), 228–9.

121 ‘La Sociedad Médica de Chile ante la última ley de Beneficencia’, *La Nación*, 19 de septiembre de 1925.

122 This discussion was not new in the political arena. In fact, it had been a recurrent concern since the creation in 1892 of a parliamentary commission to reduce the number of public employees. Diego Barría , ‘In Search for the Merit: The Academic Discussion about the Public Employees in Chile, 1884–1920’, Estudos Ibero-Americanos, 35, 2 (2009), 148–65. Efforts to advance in this direction were made in 1913, 1915, 1919 and 1923. The last of these initiatives, a commission created under Law N° 3.921, sought unsuccessfully to reduce the number of employees in each public service by 10–50 percent. ‘Reorganización de los servicios públicos’, *El Mercurio*, 10 de mayo de 1923.

123 ‘La salubridad pública’, *La Nación*, 21 de marzo de 1926.

124 Cámara de Diputados, *2° Sesión Extraordinaria*, 7 de abril de 1926, 153–6; *7° Sesión Extraordinaria*, 26 de abril de 1926, 381; *8° Sesión Extraordinaria*, 17 de abril de 1926, 412–21; *12° Sesión Extraordinaria*, 23 de abril de 1926, 575–7.

125 John D. Long, ‘The National Health Service of Chile: A Brief Synopsis of the Achievements of the Service during the Year Ended July 1, 1926’, *Public Health Reports*, 41, 27 (1926), 1571–2; *op. cit.* (note 99).

126 ‘Doctor Long: los que van a morir te saludad’, *La Nación*, 29 de junio de 1926.

127 ‘El Dr. Long no debe irse’, *La Nación*, 10 de julio de 1926; ‘La aplicación del Código Long’, *La Nación*, 11 de julio de 1926; ‘El señor Ministro de Higiene defendió ayer ante el Senado el Código Sanitario’, *La Nación*, 27 de julio de 1926.

128 ‘Los ministerios de agricultura e higiene’, *El Mercurio*, 17 de julio de 1926.

129 ‘Las economías en el Ministerio de Higiene’, *La Nación*, 12 de noviembre de 1926; ‘Numerosos empleos del Ministerio de Higiene suprimió ayer el Senado’, *La Nación*, 25 de noviembre de 1926.

130 Senado, *26° Sesión Extraordinaria*, 15 de noviembre de 1926, 563.

131 ‘¿Debe mantenerse el Ministerio de Previsión y el Trabajo?’, *El Mercurio*, 30 de noviembre de 1926.

132 Jaime Esponda, *Pablo Ramírez. El chileno desconocido* (Santiago: RIL Editores, 2013).

133 Patricio Silva, *In the Name of Reason: Technocrats and Politics in Chile* (Pennsylvania: Penn State University Press, 2008); Esponda, *op. cit.* (note 117).

134 ‘Reorganización de Servicios de sanidad’, *El Mercurio*, 11 de marzo de 1927.

135 Chile, *Boletín de las leyes y decretos del gobierno* (Santiago: Imprenta Nacional, 1927), 1042–55, 1668–71

136 ‘El subsecretario de higiene y previsión, señor Oscar Álvarez nos habla sobre la nueva organización deportiva’, *El Mercurio*, 30 de abril de 1927.

137 Quoted in Illanes, *op. cit.* (note 10), 235.

138 ‘Con la más moderna organización contara el Ministerio de Higiene’, *El Mercurio*, 25 de agosto de 1927.

139 Chile, *Ley de presupuestos de gastos generales de la administración pública* (Santiago: Imprenta Nacional, 1928).

140 Senado, *Sesión de las dos Cámaras reunidas*, 21 de mayo de 1928, 11.

141 Chile, *Ley de presupuestos de gastos generales de la administración pública* (Santiago: Imprenta Nacional, 1929).

142 Cámara de Diputados, *93° Sesión Ordinaria*, 15 de enero de 1929, 4920–2.

143 ‘Separación de Servicios del Ministerio de Bienestar Social’, *El Mercurio*, 26 de septiembre de 1930.

144 ‘La creación de una inspección de cooperativas dependiente del Ministerio de Bienestar Social’, *El Mercurio*, 17 de abril de 1930.

145 ‘Ayer se inauguró el edificio de la Dirección General de Sanidad’, *El Mercurio*, 11 de marzo de 1931.

146 Senado, *Sesión de las dos Cámaras reunidas*, 21 de mayo de 1930, 53.

147 ‘Existiría el propósito de crear dentro de poco el Ministerio de Salubridad Pública’, *El Mercurio*, 27 de junio de 1930.

148 ‘Ministerio de Bienestar Social’, *El Mercurio*, 12 de agosto de 1930.

149 Senado, *Sesión de las dos Cámaras reunidas*, 21 de mayo de 1928, 11.

150
*Ibid.*

151 Senado, *37° Sesión Ordinaria*, 12 de agosto de 1930, 829.

152 Patricio Bernedo, ‘Prosperidad económica bajo Carlos Ibáñez del Campo, 1927–1929’, *Historia*, 24 (1989), 5–105.

153 ‘Los ministerios de agricultura e higiene’, *El Mercurio*, 17 de julio de 1926.

